# Virtual magnetic resonance elastography has the feasibility to evaluate preoperative pituitary adenoma consistency

**DOI:** 10.1007/s11102-021-01129-4

**Published:** 2021-02-08

**Authors:** Kerstin Lagerstrand, Nicholas Gaedes, Stig Eriksson, Dan Farahmand, Erica De Coursey, Gudmundur Johansson, Lars Jönsson, Thomas Skoglund

**Affiliations:** 1 Medical Physics and Biomedical Engineering, Sahlgrenska University Hospital, Göteborg, Sweden; 2 Institute of Clinical Sciences, Sahlgrenska Academy, University of Gothenburg, Göteborg, Sweden; 3 Philips, Göteborg, Sweden; 4 Department of Radiology, Sahlgrenska University Hospital, Göteborg, Sweden; 5 Department of Neurosurgery, Sahlgrenska University Hospital, Göteborg, Sweden; 6 Department of Clinical Neuroscience and Rehabilitation, Institute of Neuroscience and Physiology, The Sahlgrenska Academy, University of Gothenburg, Göteborg, Sweden; 7 Department of Medicine, Sahlgrenska University Hospital, Göteborg, Sweden; 8 Department of Internal Medicine and Clinical Nutrition, Institute of Medicine, The Sahlgrenska Academy, University of Gothenburg, Göteborg, Sweden

**Keywords:** Magnetic resonance imaging, Virtual elastography, Pituitary macroadenoma, Consistency, Stiffness

## Abstract

**Purpose:**

To evaluate the use of preoperative virtual Magnetic Resonance Elastography (vMRE) for patients undergoing transsphenoidal resection of pituitary adenomas (PA).

**Methods:**

Ten patients (60.2 ± 19.6 years; 8 males) were prospectively examined with the vMRE-method prior to transsphenoidal surgery. vMRE-images, reflecting tissue stiffness were reconstructed. From these images, histograms as well as the mean stiffness values over the tumor body were extracted. Finally, vMRE-data was compared with the PA consistency at surgery blinded to vMRE.

**Results:**

In all patients, successful vMRE-examination was performed enabling evaluation of even small PAs. For tumors with homogenous tissue, the mean stiffness value increased with surgical consistency grading. For heterogenous tumors, however, the mean stiffness value did not consistently reflect the grading at surgery. On the other hand, the vMRE-images and histograms were found to be able to characterize the tumor heterogeneity and display focal regions of high stiffness that were found to affect the surgery outcome in these PAs. The vMRE-images and histograms showed great promise in characterizing the consistency at surgery for these PAs.

**Conclusion:**

Evaluation of PA consistency in preparation for surgery seems to be feasible using the vMRE-method. Our findings also address the need for high resolution diagnostic methods that can non-invasively display focal regions of increased stiffness, as such regions may increase the difficulty of transsphenoidal PA-resection.

**Supplementary Information:**

The online version of this article (10.1007/s11102-021-01129-4) contains supplementary material, which is available to authorized users.

## Introduction

Pituitary adenomas (PA) account for 10–15% of all intracranial tumors [1, 2]. The majority of the PAs have a soft texture and can be resected with curettage and suction [3]. However, about 10% of the tumors have a fibrous content that makes the tumor difficult to completely resect without injury to surrounding vital structures [4]. Preoperative knowledge of the PA consistency may alter the surgical technique and have an impact on the surgical approach and outcome, as well as reduce the risk for re-surgery or the need for post-surgical adjuvant radiotherapy [5, 6].

To characterize the consistency of PAs, various magnetic resonance imaging (MRI) methods based on standardized T1- and T2-weighted imaging, as well as contrast media enhanced MRI imaging and standardized diffusion weighted imaging have been investigated [6–10]. Up to this date, these methods have not shown sufficient feasibility in characterizing the PA consistency. Magnetic resonance elastography, however, which relies on the propagation of mechanically induced shear waves [11–13], has shown to display stiffness values that correlate with surgical grading [14]. While being a promising method for evaluation of PA consistency, the method lacks in image resolution and this may limit the visibility of small tumors and focal regions of increased tissue stiffness in larger tumors. Also, the method relies on additional hardware for induction of brain vibrations.

In this exploratory study, a novel method is proposed. Besides being a non-invasive method that relies on a clinically available MRI sequences with high speed and image resolution, this so called virtual elastography (vMRE) method is attractive for evaluation of the PA consistency due to an intrinsically high sensitivity to the viscoelastic property of the tissue. To our knowledge, however, the method has not previously been used for tumor evaluation.

Therefore, the aim of this study was to evaluate the potential usefulness of the vMRE method for preoperative evaluation of tumor consistency in patients undergoing transsphenoidal resection of PA.

## Methods

### Study cohort

The study was conducted according to the Declaration of Helsinki. Ethical approval was given by The Regional Ethics Review Board and oral and written informed consent was obtained from all participants. The data was derived from the Gothenburg Pituitary Tumor (GoPT) study, which is a prospective study that is enrolling patients scheduled for pituitary surgery at Sahlgrenska University Hospital, the sole provider of neurosurgical services for 1.8 million people in the western region of Sweden [15].

The patients were included among patients referred to the radiology department from 17/10/2018 to 31/03/2020, where patients with macroadenoma (≥ 1 cm) who underwent an MRI the day before surgery for neuro-navigation, was included in the study. The indications for surgery were (1) tumor causing chiasmal compression and/or (2) hormonal hypersecretion and/or (3) sequential imaging showing growth of the tumor.

The tumor type was verified by histology.

### Magnetic resonance imaging

All patients were examined on a 3T MRI scanner (Achieva, Philips Medical systems, The Netherlands). A standardized preoperative examination protocol, including morphological imaging in the sagittal view and standardized angiography in the transversal view (Table 1) was used in addition to diffusion weighted imaging. The full examination protocol was completed within 20 min, where the diffusion weighted imaging was completed in less than 6 min.

**Table 1:** The MRI examination protocol

Sequence name	Orientation	Field-of-View (mm^2^)	Scan matrix	Slice thickness (mm)	Number of excitations	Number of slices
3D TOF angiography	TRA	160 × 160	356 × 357	0.7	1	200
3D morphological T1-weighted imaging	SAG	250 × 250	250 × 250	1	1	183
2D diffusion weighted imaging	COR	220 × 186	124 × 88	3	5	13

*2D and 3D* two and three dimensional, *TOF* time-of-flight, *TRA* transversal, *COR* coronary, *SAG* sagittal

#### The vMRE method

The diffusion weighted imaging sequence was included in the examination protocol to characterize the viscoelastic property of the tissue using the vMRE method. For that purpose, higher b-factors (*b* = 200 and 1000 s/mm^2^) were used. With the use of higher *b*-values, the normally high sensitivity to isotropic gaussian diffusion was shifted toward non-isotropic non-gaussian diffusion and thereby, the diffusion weighted imaging sequence was made sensitive to fibrotic tissue (see le Bihan et al*.* [16] for more thorough theoretical description). To increase the contrast-to-noise-ratio and the acquisition speed of the diffusion weighted imaging, a fat-suppressed sequence based on compressed sensing (compressed SENSE reduction factor = 2) was used. Also, the sequence was based on turbo spin echo readout module to reduce geometric distortions in the sellar region from bone air cavities in the sinuses.

#### vMRE stiffness quantification

After scanning, two diffusion weighted stacks of images were automatically generated on the scanner; one for the lower *b*-value (b200 images) and one for the higher *b*-value (b1000 images). These images were then used to quantitate vMRE stiffness values. The quantification is summarized below and described in detail by le Bihan et al*.* [16]. In short, a stack of vMRE stiffness images was automatically calculated from the stack of b200 and b1000 images as the relative difference between the signal values in the b200 and b1000 images, respectively.

1 $$vMRE\,stiffness= -9.8ln\left({S}_{b200}|{S}_{b1000}\right)+14,$$

where *S*_b200_ and *S*_b1000_ are the signal values in the b200 and b1000 images and the scaling factor of − 9.8 and the shift factor of 14 were previously determined in a calibration step by le Bihan et al. [16]*.*

**Fig. 1:**
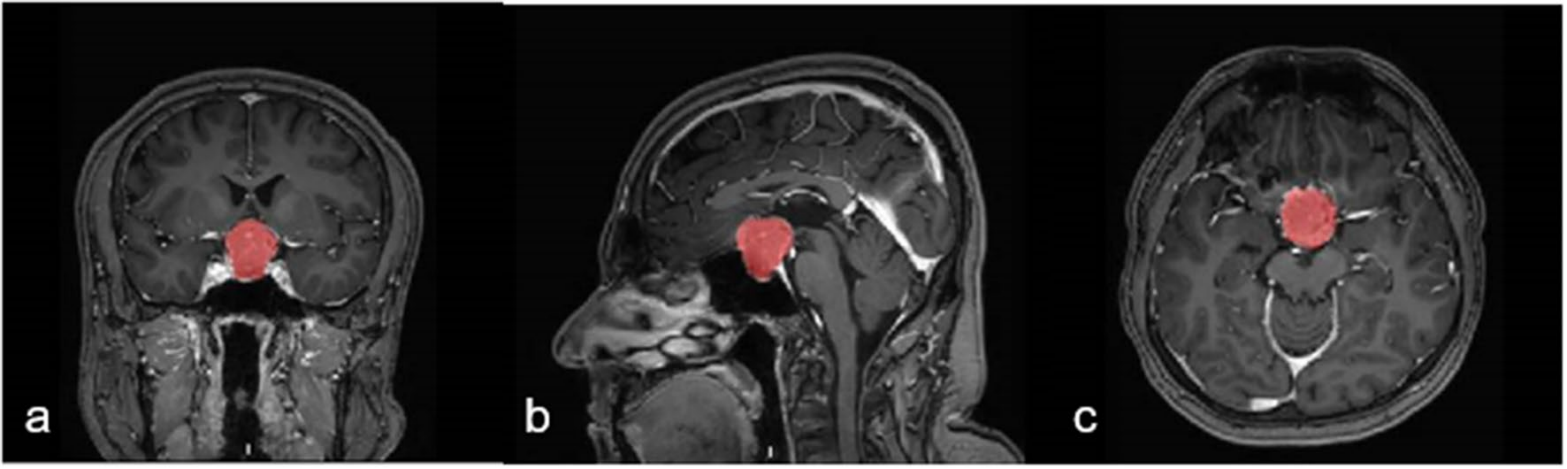
Illustration of the tumor ROI on T1-weighted images in the coronal, sagittal and transversal plane

Then, the tumor region was segmented on all vMRE stiffness images that included the tumor body. The border of the segmentation was located approximately one pixel away from the visible edge of the tumor, ensuring that no background tissue was included in the tumor region (Fig. 1). Also, pixels in major blood vessels were excluded from the region. The segmentation was performed manually by two trained observers (E.D.C and N.G), unaware of the surgical findings, and took approximately 5 min per individual. N.G repeated the segmentations to determine the intra-observer agreement. The inter-observer agreement between the observers was also determined.

From the segmented tumor regions, the mean vMRE stiffness and the standard deviation of the mean were automatically extracted within 1 sec. Also, histograms were automatically extracted to visualize the distribution in vMRE stiffness values within the tumor region.

The quantification of the vMRE stiffness was performed off-line using MATLAB R2016a (Mathworks, Natick, Massachusetts, USA).

#### The PA consistency at surgery

All surgeries were performed by one of two surgeons (D.N. or T.S.). The surgeons, blinded to the vMRE stiffness images, gave a single classification of tumor stiffness in each case as very soft, soft, moderate, hard or very hard (I–V). The system for classification of the consistency was jointly developed by the surgeons and has systematically been performed as a part of the prospective GOPT-study since 2015. In addition, the surgeons also made separate notes about the consistency of the tumor in free text.

#### Descriptive and statistical analysis

Categorical variables were expressed by numbers and continuous variables by mean ± SD.

The vMRE stiffness images and histograms, as well as the quantitative parameters (mean vMRE stiffness and the standard deviation of the mean) were compared with surgical findings as well as between individuals.

The Chi-squared test was conducted to compare vMRE-values and ADC-values between surgical gradings at *p* = 0.05 significance level.

The inter-observer agreement was determined using intra-class correlation coefficients (ICC) with 95% confidence intervals, where the two-sided random ICC model was used to determine the consistency of the agreement.

The statistical analyses were performed using the MatLab software.

## Results

### Patient characteristics and PA consistency at surgery

Ten patients (60.2 ± 19.6 years; 8 males) underwent preoperative MRI examination before endoscopic transsphenoidal resection of PA. The patient characteristics and results on PA consistency at surgery are shown in Table 2. The patients displayed a large range of tumor size from 10 to 44 mm and a large variation in PA consistency from very soft to very hard. Results from 6-months post-op follow-up, included when available, showed large regions of tumors residuals in two patients (ID = 1 and 7). Also, in patients 2 and 3, small regions of tumor residuals were found at 6-months post-op follow-up.

**Table 2:** Patient characteristics and results for all patients included in the study

ID	Gender	Age (years)	Tumor size (mm)	Tumor type	Tumor texture	Tumor consistency grade	Surgery	6 months post-op	Mean ± SD vMRE stiffness	Appearance on vMRE image	vMRE histogram distribution	Heterogenous on MRI
1	W	29	32	Hypophysis adenoma GH producing adenoma	Streaky	V	Primary	Tumor residuals	10.5 ± 0.8	Low variation in color depth, only high stiffness values	Narrow from 8–12, not skewed	No
2	M	81	29	Hypophysis adenoma NFPA	Homogenous	IV	Primary	GTR	7.8 ± 1.3	Variation in color depth, higher stiffness values close to the point of entry	Moderately wide from 5–10, highly skewed right	Yes
3	M	40	10	Hypophysis adenoma NFPA	Streaky, heterogenous	IV	Re-op	Small amount of tumor residuals	9.4 ± 1.1	Variation in color depth, towards higher stiffness values	Wide from 6–12, skewed right	Yes
4	M	86	40	Hypophysis adenoma NFPA	Partly streaky, heterogenous	III	Primary	np	8.5 ± 1.3	No focal regions but patchy appearance	Wide from 4.5–11.5, not skewed	Yes
5	M	80	33	Hypophysis adenoma NFPA	Homogenous	I	Primary	GTR	8.2 ± 1.0	Intermediately stiff to the left and soft to the right	Intermediately wide from 5–10, not skewed	Yes
6	M	65	24	Hypophysis adenoma NFPA	Initially streaky then solid	IV	Re-op	GTR*	6.9 ± 1.2	Focal region with high stiffness at point of entry	Wide from 4–10, slightly skewed right	Yes
7	M	46	30	Hypophysis adenoma NFPA	Streaky caudally at frontal region	IV	Primary	Large amount of tumor residuals	7.2 ± 1.4	Focal region with high stiffness at point of entry	Wide from 4–10, not skewed	Yes
8	M	74	18	Hypophysis adenoma NFPA	Homogenous	II	Primary	GTR	8.3 ± 1.2	Lower stiffness values close to the point of entry, higher stiffness at deeper locations	Wide 6–11, skewed right	Yes
9	M	37	44	Hypophysis adenoma NFPA	Homogenous	II	Primary	GTR	7.4 ± 0.9	Low variation in color depth, intermediate stiffness values	Narrow from 5–9, not skewed	No
10	F	64	29	Hypophysis adenoma NFPA	Homogenous	II	Primary	GTR	9.0 ± 1.0	Higher stiffness values close to the point of entry	Wide from 5 to 11, highly skewed right	Yes

Tumor size represents the largest dimension of the tumor body detected in the image stack. *W* woman, *M* male, *NFPA* clinically non-functioning adenoma, *re-op* reoperation, not performed, *vMRE* virtual magnetic resonance elastography, *SD* standard deviation, *GTR* Gross total resection

^*^post op MRI performed the day after surgery

### Quantification of vMRE stiffness values

The diffusion weighted imaging was successfully performed in all patients, resulting in vMRE stiffness images with high image quality.

**Fig. 2:**
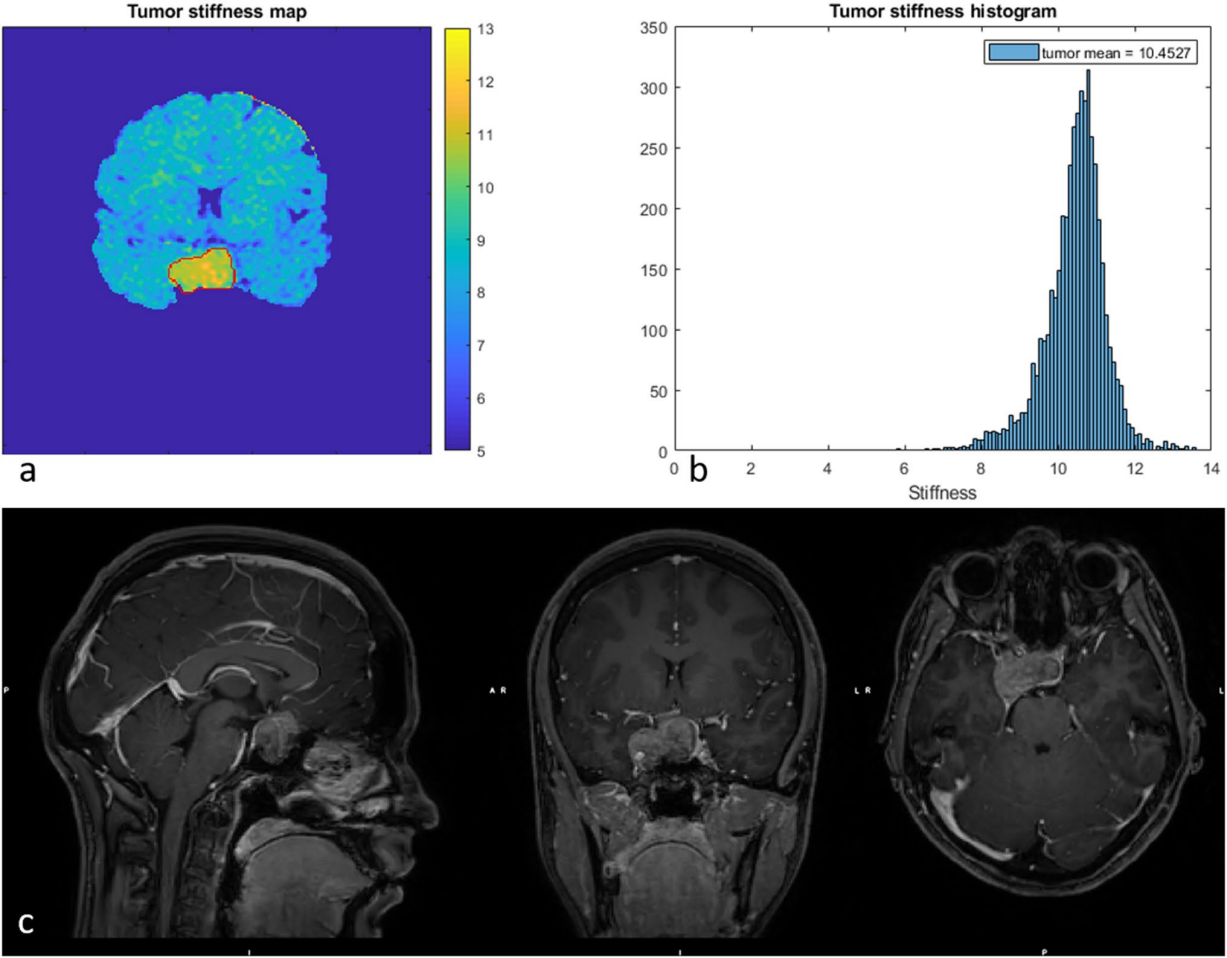
The vMRE stiffness image (above left) and histogram (above right) for PA with ID 1 displayed, in consensus with PA consistency at surgery, very high stiffness with low variation over the tumor body

Overall, the mean vMRE stiffness did not differ between groups of patients with different grading of PA consistency at surgery (Table 2). However, the mean vMRE stiffness value increased with increased grading for homogenous PAs (Fig. 2), displaying low color depths in the vMRE stiffness images and more narrow histogram distributions.

**Fig. 3:**
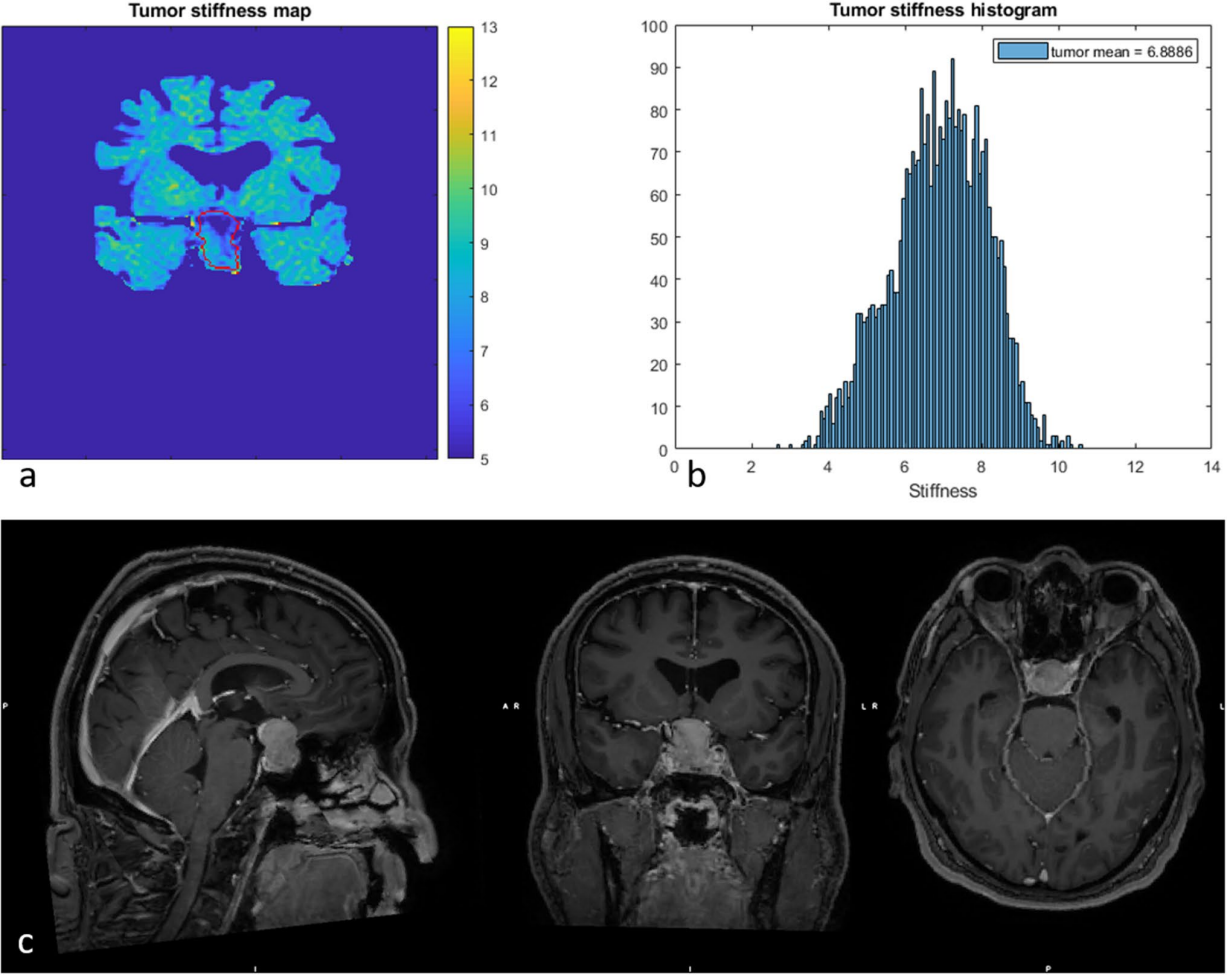
The vMRE stiffness image (above left) for PA with ID 6 displayed a region of much higher stiffness values in lower part (close to the point of entry of the surgery) compared with the upper part of the tumor body. Corresponding histogram (above right) enhanced the tumor heterogeneity as a large spread in stiffness values and a slightly skewed distribution

**Fig. 4:**
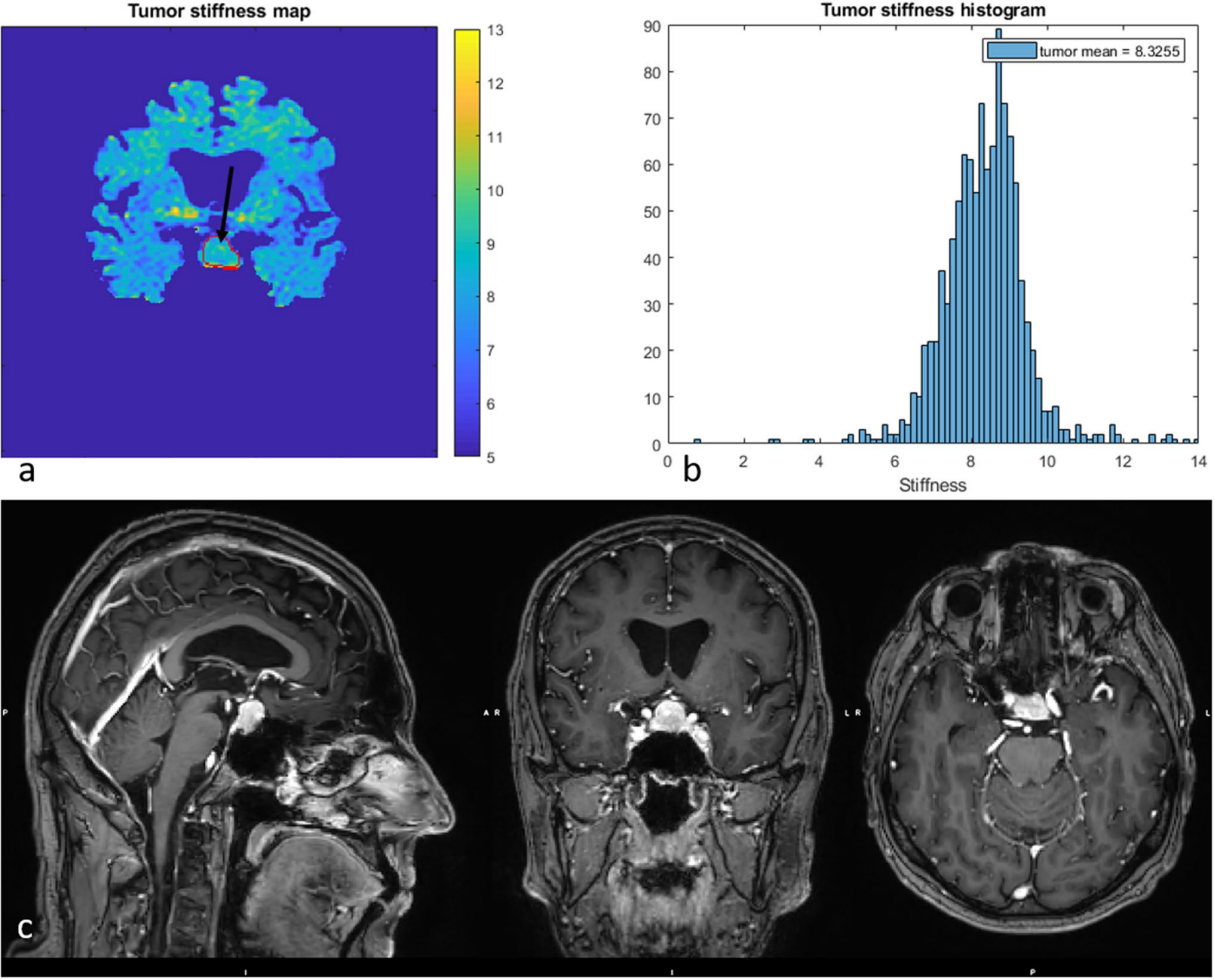
The vMRE stiffness image (above left) for PA with ID 8 displayed a region of higher stiffness values deeper into the tumor body (black arrow) and lower stiffness values close to the point of entry. Corresponding histogram (above right) enhanced the tumor heterogeneity as a slightly skewed distribution. The high stiffness values at the very bottom outside the tumor outline are likely bone

**Fig. 5:**
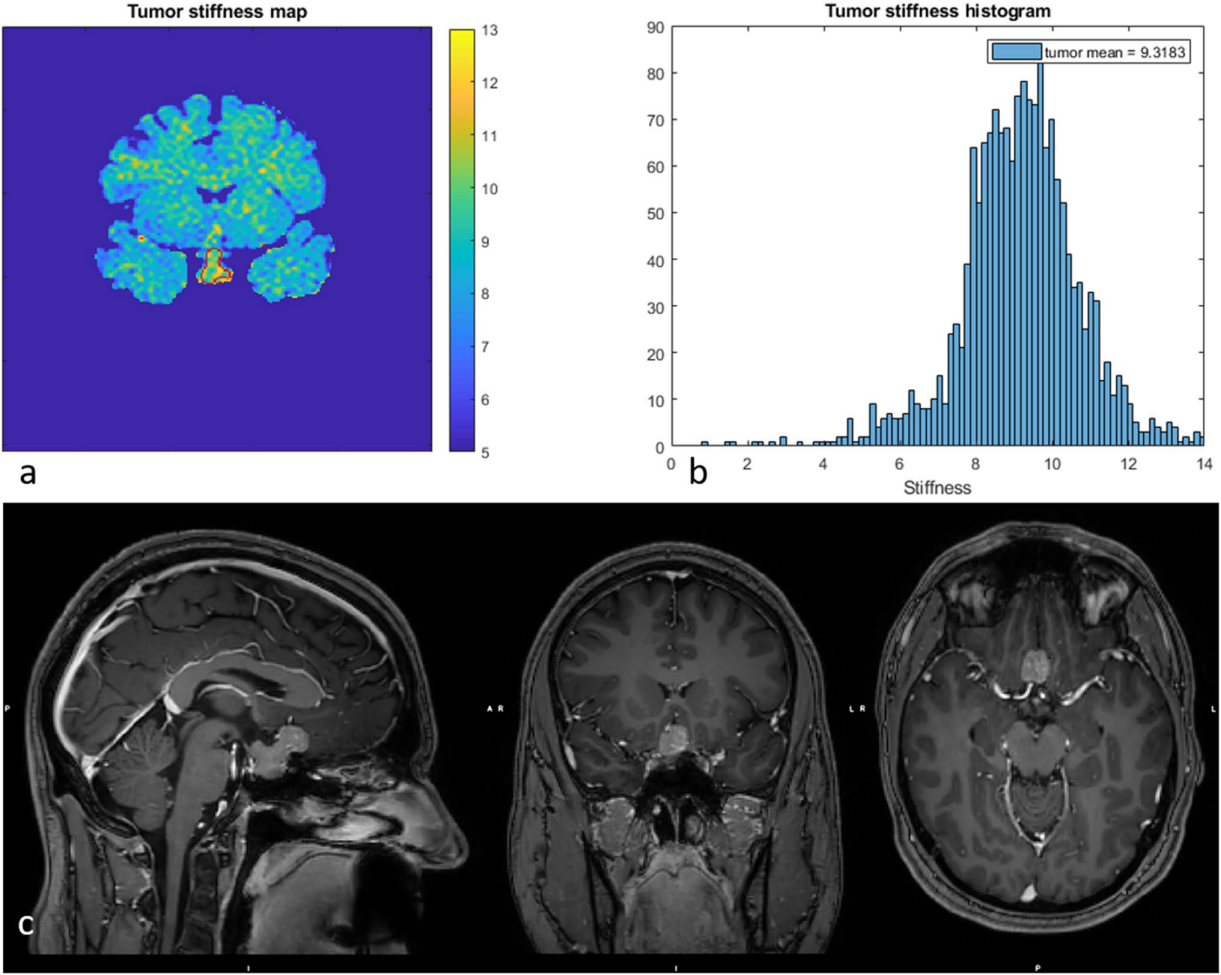
The vMRE stiffness image (above left) and histogram (above right) for PA with ID 3 displayed a stiff and very heterogeneous tissue texture with a skewed histogram and a wide variation of stiffness values over the tumor body

**Fig. 6:**
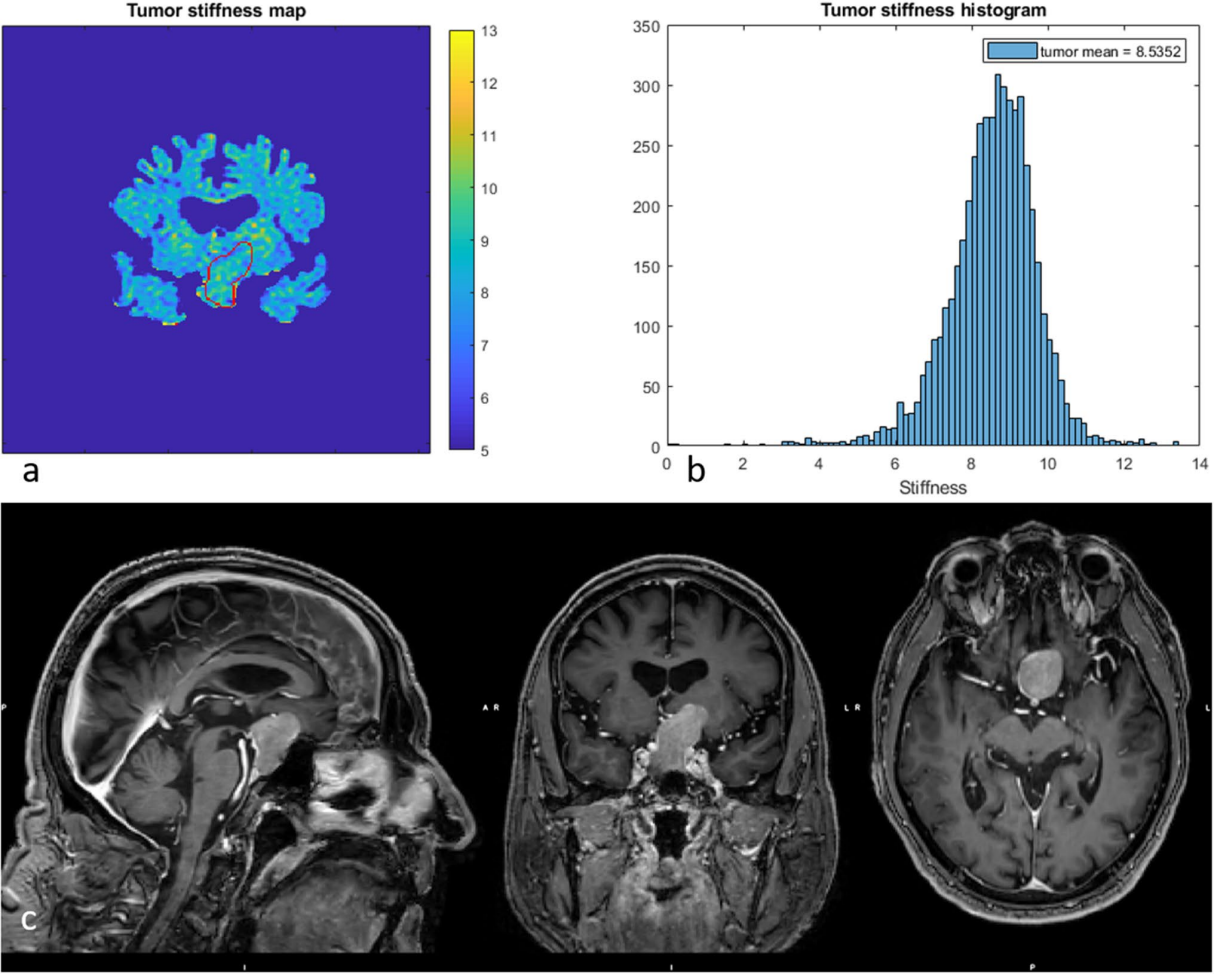
The vMRE stiffness image (above left) and histogram (above right) for PA with ID 4 displayed a wide variation between image slices in the anterior posterior direction from high to low stiffness values. The chosen slice shows a stiffer part of the tumor

**Fig. 7:**
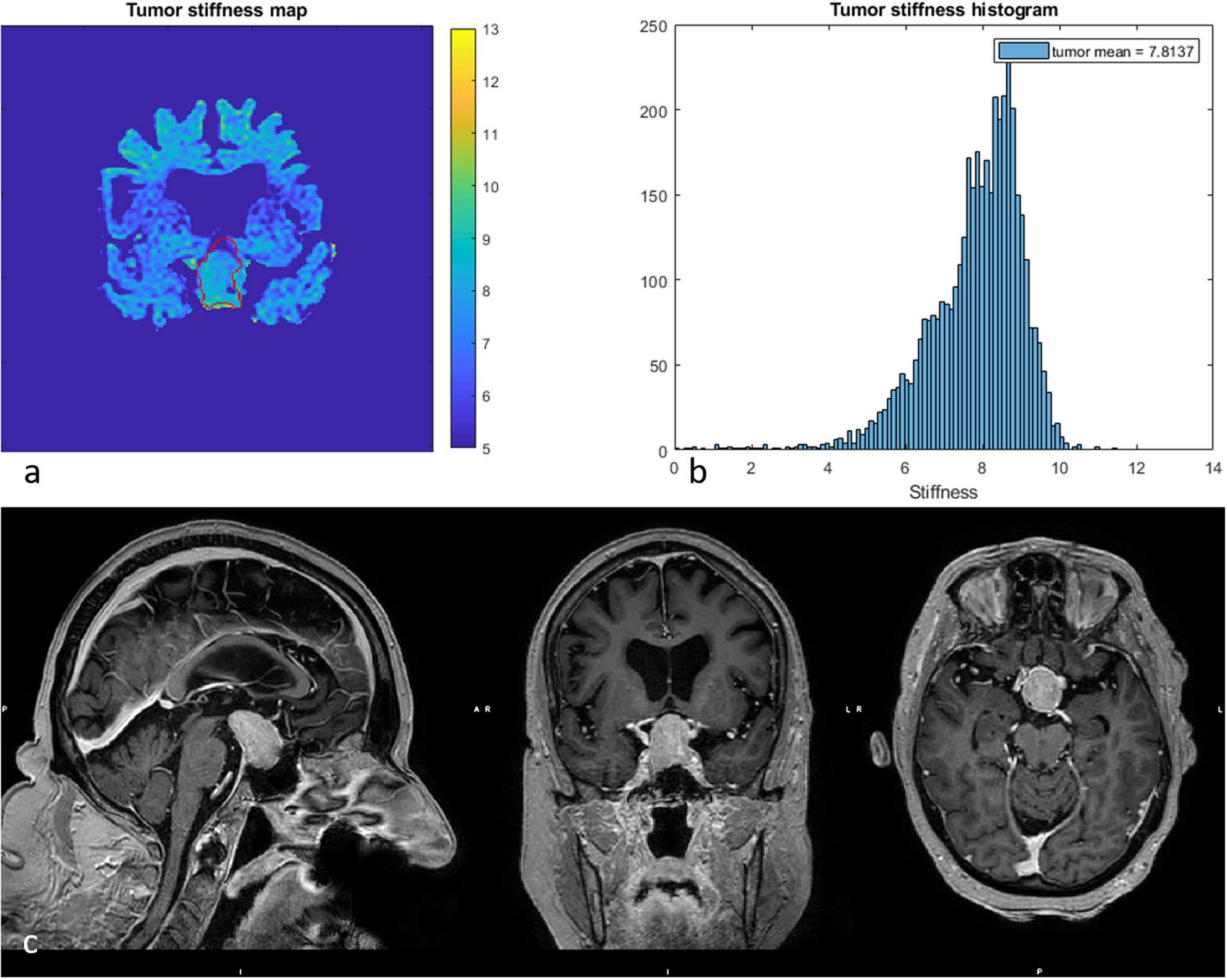
The vMRE stiffness image (above left) and histogram (above right) for ID 2 showed a highly skewed histogram with a large proportion of high stiffness values, especially in the lower part of the tumor body where the incision was made. Interestingly, the very top part of the tumor is shown to be very soft

Between but also within individuals, the PAs displayed a large variation in vMRE stiffness values. Some PAs displayed focal regions of increased tissue stiffness, depicted as clusters of pixels with higher values in the vMRE stiffness images. The histograms characterized such heterogeneity as right-skewed distributions while streaky tissue was characterized in histograms as a large distribution of stiffness values. For example, ID 6 that was re-operated showed a region of higher stiffness values around 8.5 in the lower part of the tumor (close to the point of entry of the surgery) while deeper into the body, the tumor appeared soft with low stiffness values around 4 (Fig. 3). The corresponding histogram visualized the tumor heterogeneity as a large spread in stiffness values and a slightly right-skewed distribution. The histogram of ID 8 also displayed a heterogeneous PA with a right-skewed distribution of stiffness values. The corresponding vMRE stiffness image of ID 8 displayed a PA with low stiffness values around 4 close to the point of entry of surgery, while small regions of higher stiffness values, around 9, were found deeper into the tumor body (Fig. 4). In this case, the PA was graded as soft at surgery and showed gross total resection on post-op MRI. Both the vMRE stiffness image and histogram for ID 3 displayed the PA tissue as stiff and heterogeneous (Fig. 5), in similar with the PA consistency grading and tumor texture (Table 2). For ID 4, the vMRE stiffness images displayed the PA as intermediately stiff with a wide variation between image slices in the anterior posterior direction from high to low stiffness values (Fig. 6). The corresponding histogram also displayed a wide distribution of stiffness values. This PA was found to be soft/moderate by the consistency grading (Table 2). For ID 2, the PA appeared to be very heterogenous in the vMRE stiffness images and showed a right-skewed histogram with a large proportion of high stiffness values. At surgery, the PA was graded as hard but showed gross total resection on post-op MRI (Fig. 7). See supplementary figures for remaining PAs.

The ICC analysis showed excellent agreement (*r* = 1.00 and *p* < 0.001) with near identical mean vMRE stiffness values in all three cases (E.D.C, N.G1, N.G2).

## Discussion

In this study, a non-invasive diagnostic method for evaluation of PA consistency is proposed that has the potential to improve the risk assessment and surgical planning of these patients. For homogenous tumors, the so called vMRE method showed, in consensus with surgery grading, increased mean stiffness values with increased PA consistency. Moreover, stiffness values displayed in color in vMRE stiffness images and histograms greatly improved the tumor characterization, emphasizing focal regions of increased tissue stiffness that may pose a problem during surgery.

While most PAs have soft texture and can be resected easily, a small proportion are hard and are more difficult to dissect from critical surrounding structures. Such cases may add substantial amount of operative time and may demand further surgical methods with associated risks. Improving the preoperative prediction may lead to better counseling of the risks and benefits for the patient. Hence, new diagnostic tools are warranted for preoperative evaluation of PA consistency.

Several studies have used a variety of MRI-based methods to find correlations between image characteristics and PA consistency. Contrast media enhanced MRI has been used, without success, to differentiate soft from hard tumors using intratumorally hyperintense dots [10]. Also, no correlation between T2-weighted image characteristics and PA consistency has been found [17]. A few studies have correlated standardized diffusion weighted imaging to PA consistency [8, 18], but found that the calculated diffusion value is not predictive of tumor consistency [19]. This might, to some part, be assigned to perfusion that inherently contaminates the diffusion value. Since perfusion can vary greatly between tumors and, thus, introduce individual biases in the measurement, the method is not suitable for evaluation of underlying viscoelastic property of the PA tissue. Moreover, standardized diffusion weighted imaging is based on fast but susceptibility-sensitive EPI read-out sequences that are unsuitable for imaging near bone and air cavities and, as such, may contribute artifactual signals to the PA evaluation.

Present method showed promise in characterizing the PA consistency at surgery. The method relies on non-standardized diffusion weighted imaging that utilizes a robust read-out to reduce the artifactual distortion of tissues close to bone air cavities. By introducing the so-called turbo spin echo read-out into the diffusion weighted imaging sequence, high quality images could be obtained without visible artefacts in the sellar region. This further improved the PA evaluation. The promising findings are to some part attributed to the performance of the MRI sequence, but mainly due to the introduction of higher *b*-values and, thus, a reduced sensitivity to perfusion with increased sensitivity to non- Gaussian diffusion. Non-Gaussian diffusion is believed to arise from diffusion barriers, such as cell membranes in fibrotic tissue. Hence, vMRE method could potentially provide a better understanding of microstructural tissue changes in PAs associated with disease pathology and enable fast and detailed preoperative evaluation of the tumor stiffness.

A recently published study has utilized standardized magnetic resonance elastography to determine PA consistency at surgery, where a correlation between the stiffness values and consistency grading was reported [14]. As in present study, the included sample size was small (*n* = 10) but the results were encouraging, suggesting that stiffness have the possibility to predict PA surgery outcome. The considerably higher resolution, but also the shorter scan time and availability of the proposed method makes the proposed method an attractive alternative to the standardized magnetic resonance elastography method for evaluation of PA consistency. Also, the present method does not rely on additional hardware for induction of measurable shear wave vibrations.

In similar to the standardized magnetic resonance elastography method, the vMRE method seemed to offer mean stiffness values that increased with surgical grading for homogenous tumors. The high-resolution vMRE method also offered detailed information about the tumor tissue in terms of color vMRE stiffness images and histograms, where our findings showed the importance of characterizing tumor tissue heterogeneity in detail. Focal regions of tissue with higher stiffness may alter the surgical approach and have an impact on the surgical outcome. For example, one of the PA patients (ID 6) was re-operated and as a result displayed a region of very stiff values due to scarring, clearly displayed in the vMRE stiffness images at the point of surgical entry. This might have limited the removal of the tumor. Preoperative knowledge of the position of the focal region might have altered the course of the surgery in this subject. Future studies are encouraged to confirm the diagnostic value of the vMRE method and the clinical usefulness of this novel stiffness contrast.

Since the proposed method has not previously been used for patients undergoing transsphenoidal resection of PAs, there is a lack of knowledge regarding the appearance of the PA tumors in the vMRE stiffness images and histograms, associated to the surgeon’s experience at surgery. For homogenous PAs, there seems to be a consistency between the mean vMRE stiffness value and the surgery grading. For heterogenous tumors, however, deeper knowledge seems to be needed, as exemplified by ID 4. While the consistency of most PAs was found to be successfully characterized with the present method, this softer PA displayed a mean vMRE stiffness value in similar to PAs with high consistency grading. No focal regions of higher stiffness values were seen in the vMRE stiffness images. However, the histogram displayed a wide distribution of stiffness values, representing a patchy heterogenous texture pattern. Such texture pattern might give a soft experience at surgery.

### Limitations

Being a feasibility study, a small number of patients were included in the evaluation and, as such, this limits the strength of the conclusion. Future investigations on larger study cohorts and different types of adenomas are therefore encouraged to confirm our findings and evaluate the predictive value of the vMRE method for surgical PA outcome.

The proposed vMRE-method utilized a diffusion weighted imaging sequence that was sensitized to enhance tissue textures with dense diffusion barriers, such as fibrotic tissue. For that purpose, high *b*-values were used. Even though present findings show great promise, we cannot neglect the fact that the vMRE-method might have benefitted from even higher *b*-values, however, at the expense of increased spatial resolution or extended scan time.

In similar to the work by Hughes et al*.* [14], the study design was standardized so that each case was assigned a single classification of surgical impression of PA consistency, using a 5-point scale from very soft to very hard. However, the vMRE method seems to be able to characterize tumor heterogeneity in detail and our findings show the importance of including such information in the PA evaluation. To fully investigate the diagnostic value of the vMRE method, future studies should preferably include the surgeon’s impression of heterogeneity in the classification protocol.

The vMRE stiffness calculation used in this study is straightforward, where the mean tumor stiffness can be estimated directly from the mean signals of the b200 and b1000 images. Future MR scanner releases may implement the proposed method and offer automatic evaluation. Today, however, an off-line software is needed to calculate the vMRE stiffness on a pixel by pixel basis and reconstruct vMRE stiffness images and histograms for PA heterogeneity evaluation. This may limit the evaluation in centers with no technical/programming competence.

## Conclusions

Present study showed high feasibility of the vMRE method for preoperative evaluation of the PA consistency. The vMRE-method produced artifactual-free images of high quality and resolution, enabling evaluation of tumor heterogeneity. Also, the mean vMRE stiffness value was found to increase with surgical stiffness grading for solid tumors with homogenous texture. Further studies are encouraged to confirm our findings and determine whether the vMRE method provides useful information in the preoperative planning of PAs.

## Supplementary Information

Below is the link to the electronic supplementary material.

Figure S1. The vMRE stiffness image (above left) and histogram (above right) for PA with ID 7 displayed a small focal region with higher stiffness values around 10 point of entry of the surgery. (PNG 66 kb)

Figure S2. The vMRE stiffness image (above left) and histogram (above right) for PA with ID 9 displayed in consensus with surgent consistency grading, the PA consistence as homogenously soft with low variation in stiffness values over the tumor body. (PNG 84 kb)

Figure S3. The vMRE stiffness image (above left) and histogram (above right) for PA with ID 10 displayed a homogenously stiff tumor. At surgery, this tumor was graded as soft but left residual tumor tissue, seen on the 6 months follow-up MRI. (PNG 77 kb)

Figure S4. The vMRE stiffness image (above left) and histogram (above right) for PA with ID 5 displayed a heterogenous PA with low stiffness and a variation in stiffness from left to right. At surgery, this tumor was graded as very soft. (PNG 67 kb)

## Data Availability

All images are available on request.

## Code availability

The off-line post-processing software code is available on request.
