# Supplementary figures and images for: Virtual magnetic resonance elastography has the feasibility to evaluate preoperative pituitary adenoma consistency
Source: Pituitary. 2021 Feb 8;24(4):530–41. doi: 10.1007/s11102-021-01129-4 (PMC8270838; doi:10.1007/s11102-021-01129-4)

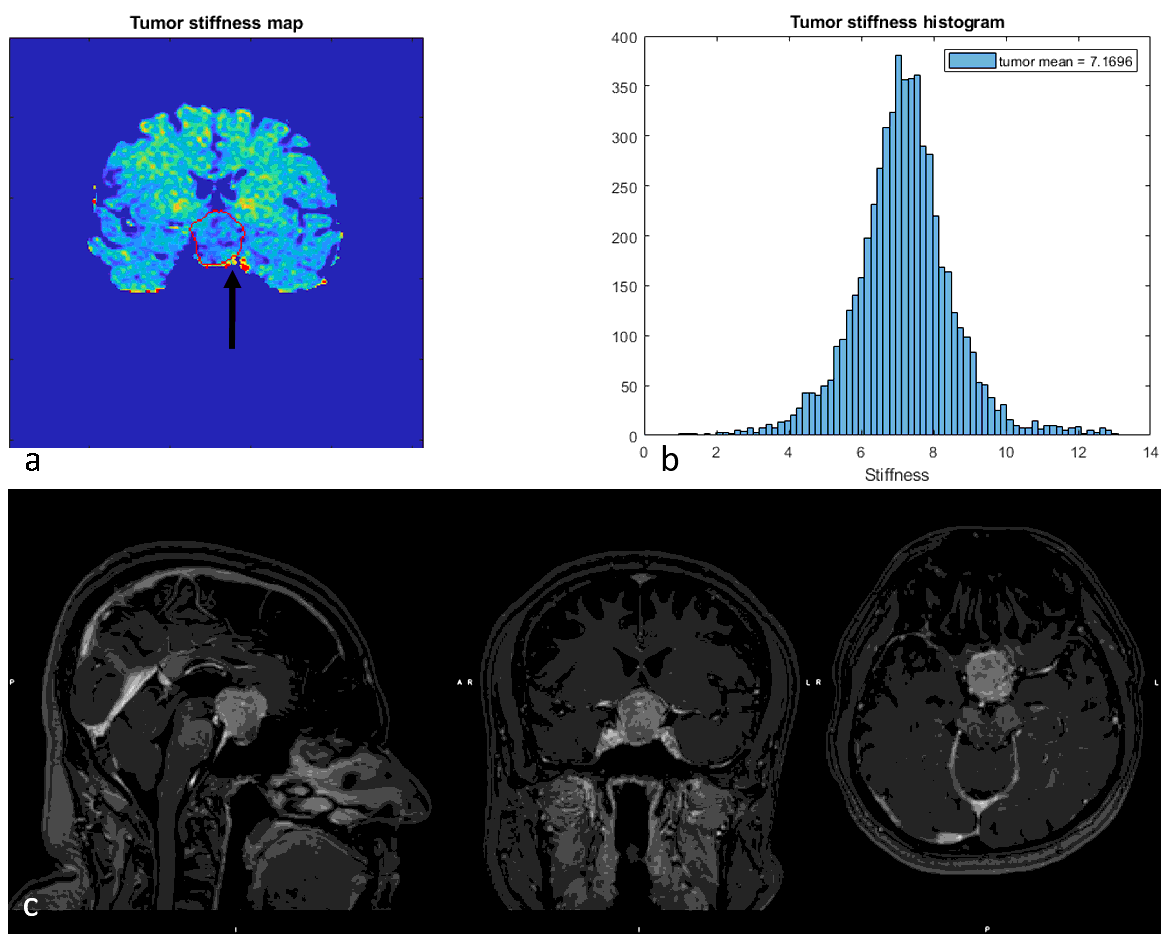

Supplement: Supplementary file 1 — Figure S1. The vMRE stiffness image (above left) and histogram (above right) for PA with ID 7 displayed a small focal region with higher stiffness values around 10 point of entry of the surgery. (PNG 66 kb) [file 11102_2021_1129_MOESM1_ESM.png]

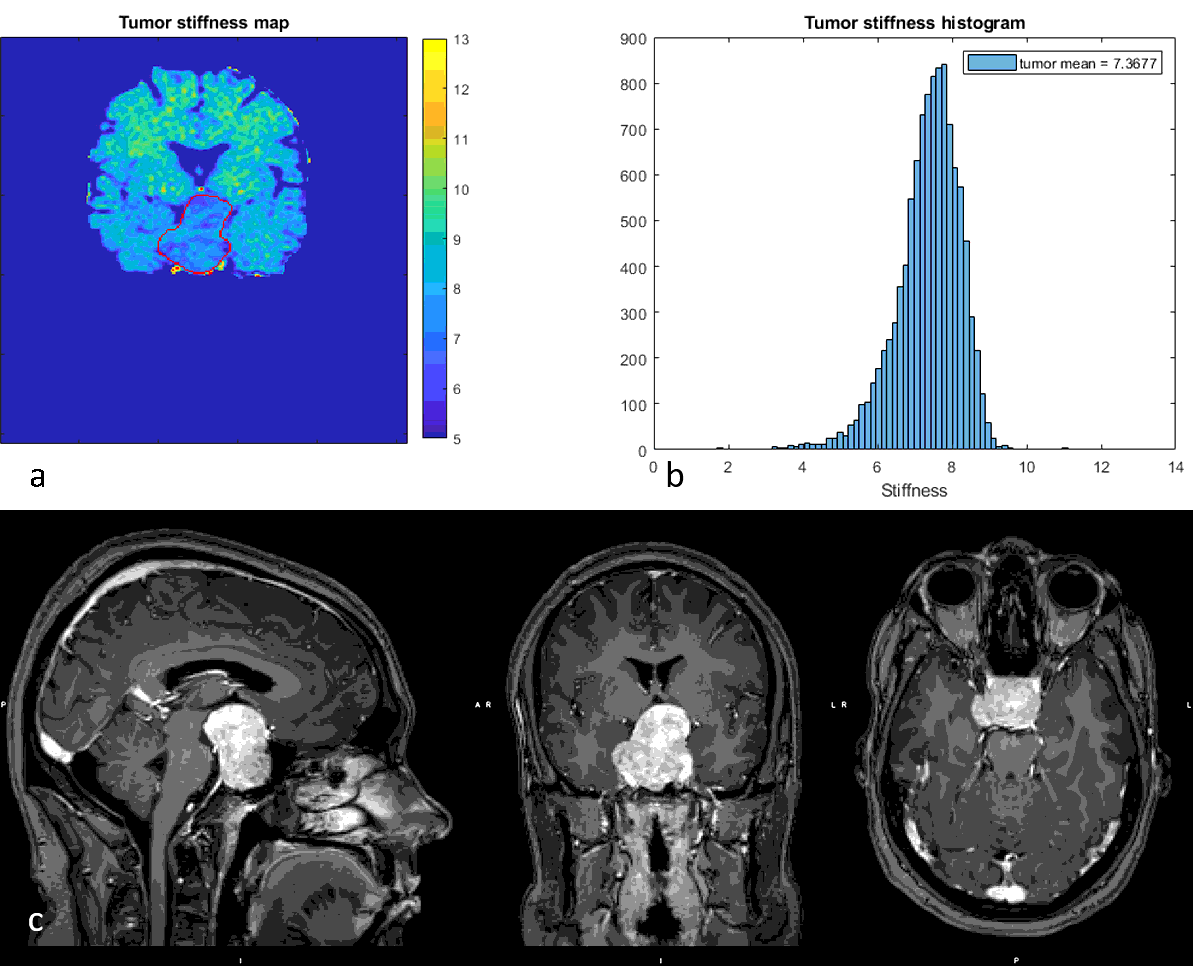

Supplement: Supplementary file 2 — Figure S2. The vMRE stiffness image (above left) and histogram (above right) for PA with ID 9 displayed in consensus with surgent consistency grading, the PA consistence as homogenously soft with low variation in stiffness values over the tumor body. (PNG 84 kb) [file 11102_2021_1129_MOESM2_ESM.png]

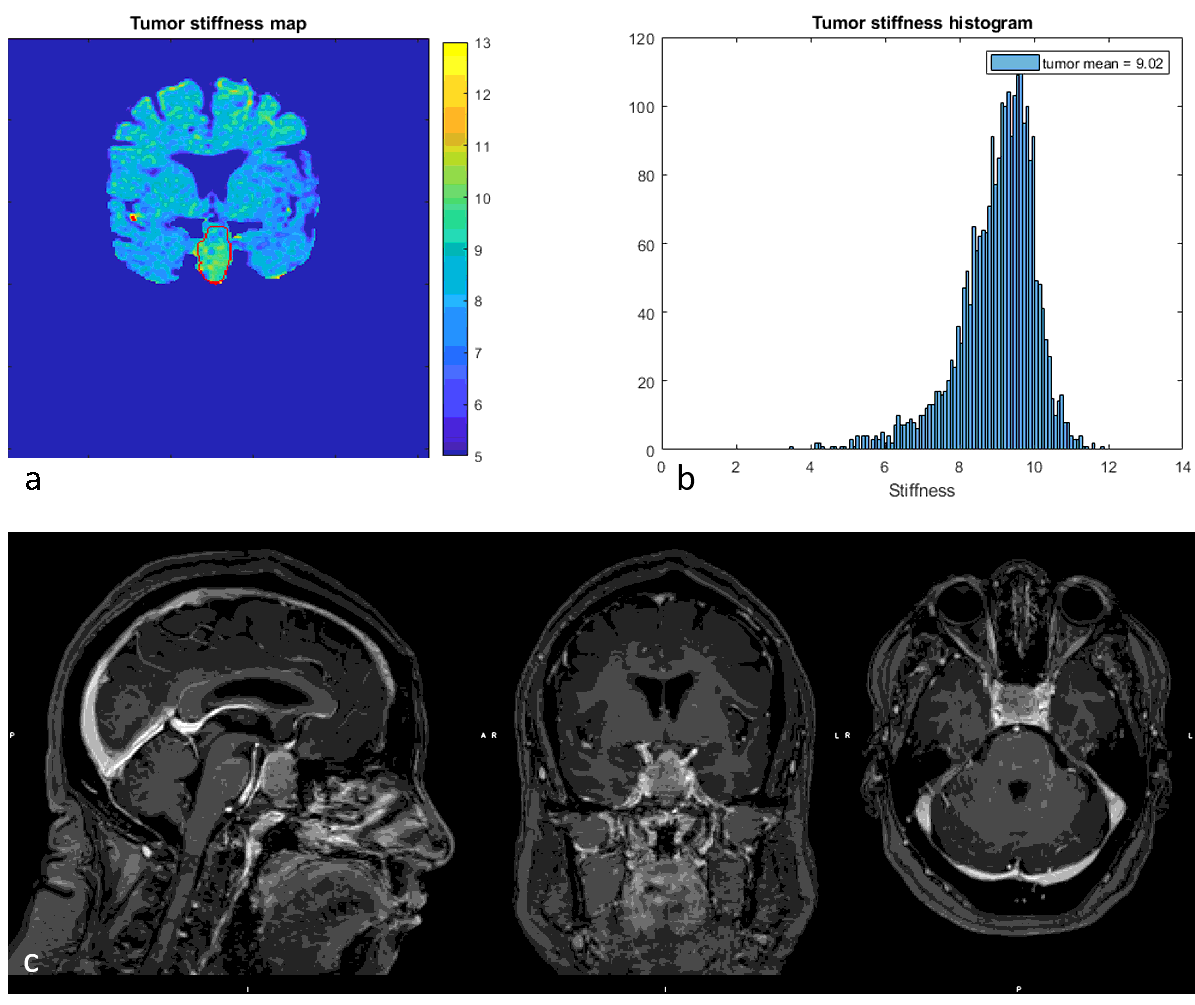

Supplement: Supplementary file 3 — Figure S3. The vMRE stiffness image (above left) and histogram (above right) for PA with ID 10 displayed a homogenously stiff tumor. At surgery, this tumor was graded as soft but left residual tumor tissue, seen on the 6 months follow-up MRI. (PNG 77 kb) [file 11102_2021_1129_MOESM3_ESM.png]

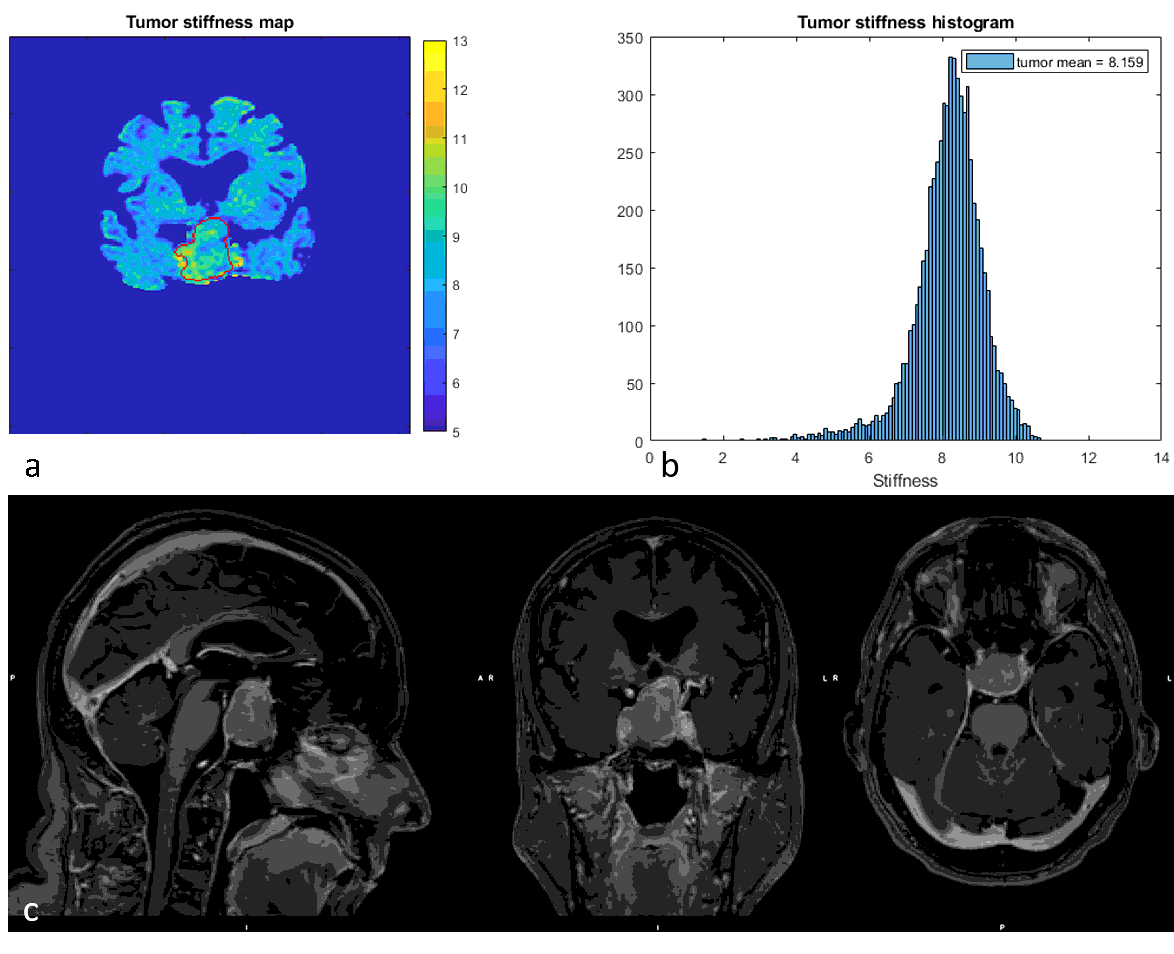

Supplement: Supplementary file 4 — Figure S4. The vMRE stiffness image (above left) and histogram (above right) for PA with ID 5 displayed a heterogenous PA with low stiffness and a variation in stiffness from left to right. At surgery, this tumor was graded as very soft. (PNG 67 kb) [file 11102_2021_1129_MOESM4_ESM.png]
